# Supplementary material for: Genomic Characterization of a Relative of Mumps Virus in Lesser Dawn Bats of Southeast Asia
Source: Viruses. 2023 Feb 28;15(3):659. doi: 10.3390/v15030659 (PMC10053730; doi:10.3390/v15030659)
Supplement: Supplementary file 1 [file viruses-15-00659-s001.zip › viruses-2184600-supplementary.pdf]

viruses

MDPI

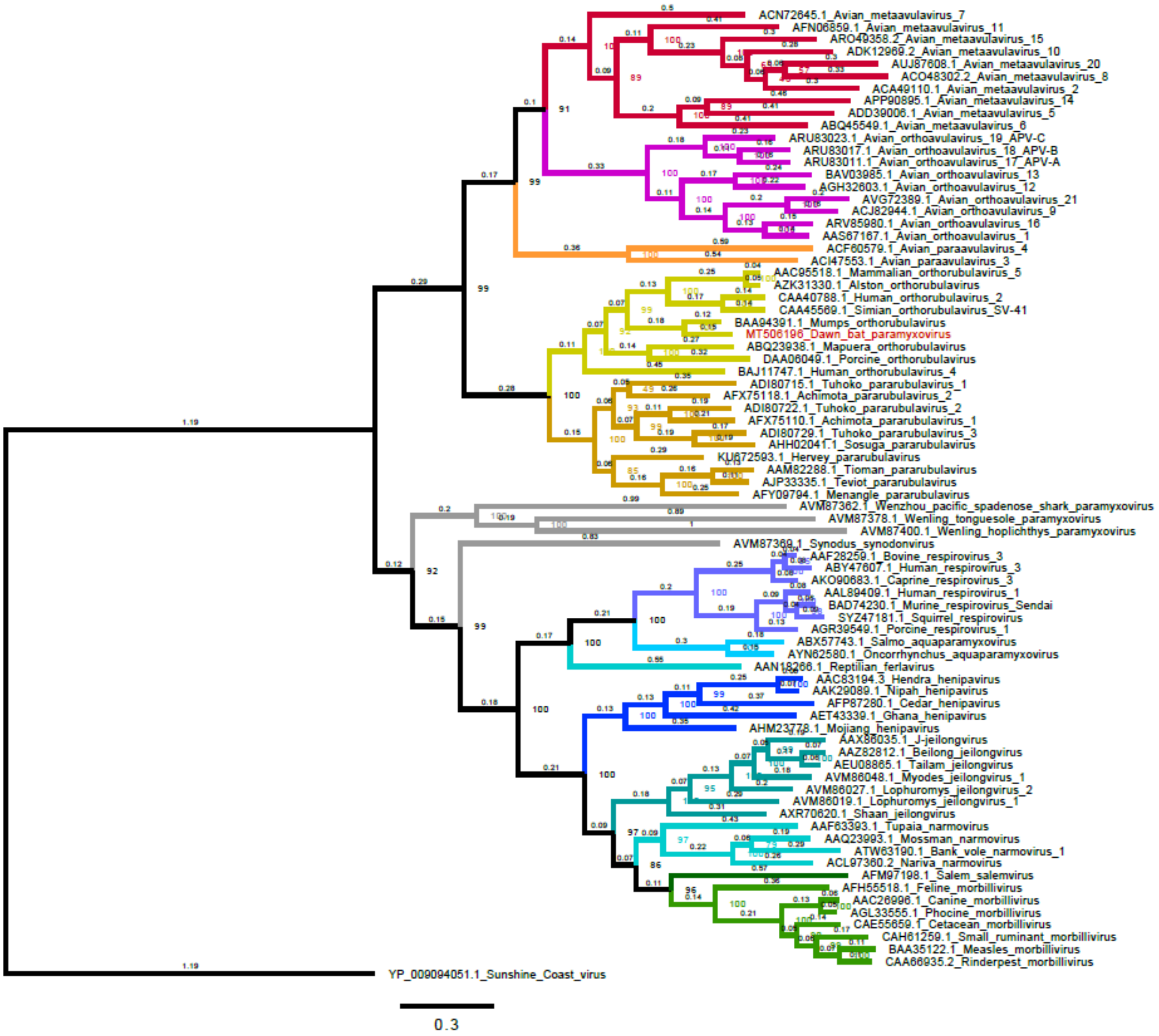

Supplemental Figure S1. Tree submitted to ICTV for demarcation of DbPV. Demarcation criteria for *Paramyxoviridae* is defined primarily by tree topology, requiring a branch length between the nearest node and the tip of the branch to be 0.03 in a tree generated as described in the ICTV's public information. The parameters described in ICTV guidance were followed and sequences for the alignment obtained from the ICTV Resources page. Parameters require complete L protein amino acid sequences of members of the family *Paramyxoviridae* (we used the sequences included in ICTV's example). The following parameters were followed using MEGA X [40] with the partially-complete L sequence from DbPV and the L sequences provided by ICTV as written in the ICTV's guidance: "Complete L protein amino acid sequences were aligned by Clustal W with gap generation penalties of 5 and extension penalties of 1 in both multi and pairwise alignments. The evolutionary history was inferred by using the Maximum Likelihood method and JTT matrix-based model" with 1,000 bootstrap replicates [41] using IQ-TREE [15]. The highest log likelihood was: -262121.051. The tree was visualized using FigTree, labeled with bootstrap values and branch lengths, and color coded to match the genus labels according to ICTV's example [17]. This analysis involved 79 amino acid sequences. There were a total of 2,746 positions in the final dataset. The branch length between the nearest node and the tip of the DbPV branch is 0.15, label shown in red.

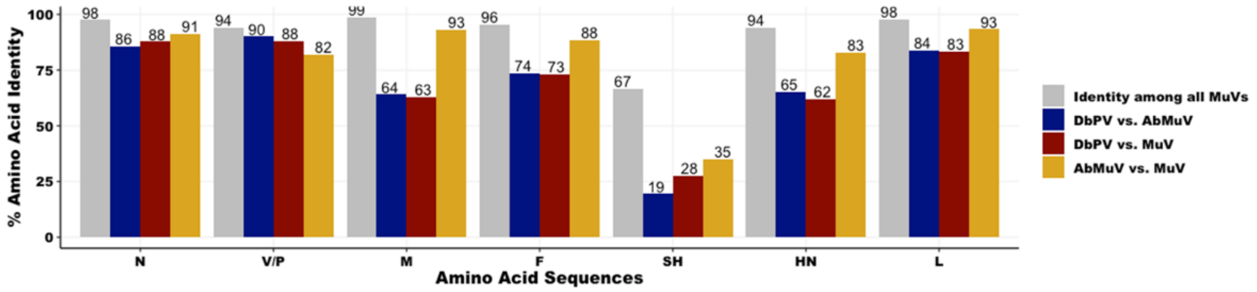

Supplemental Figure S2. Comparison of amino acid identity among MuV, AbMuV, and DbPV. The percent amino acid identity is shown along the y-axis for each amino acid sequence, labelled along the x-axis. The bars represent the minimum percent amino acid identity of overlapping alignment positions where the sequences agree among MuVs (grey), between DbPV and AbMuV (HQ660095; blue), between DbPV and MuV (NC_002200; maroon), and between AbMuV and MuV (HQ660095 and NC_002200; gold). Note that the calculations included partially complete amino acid sequences for some of the DbPV gene products and from the public databases for human MuVs, which is a limitation of this comparison.

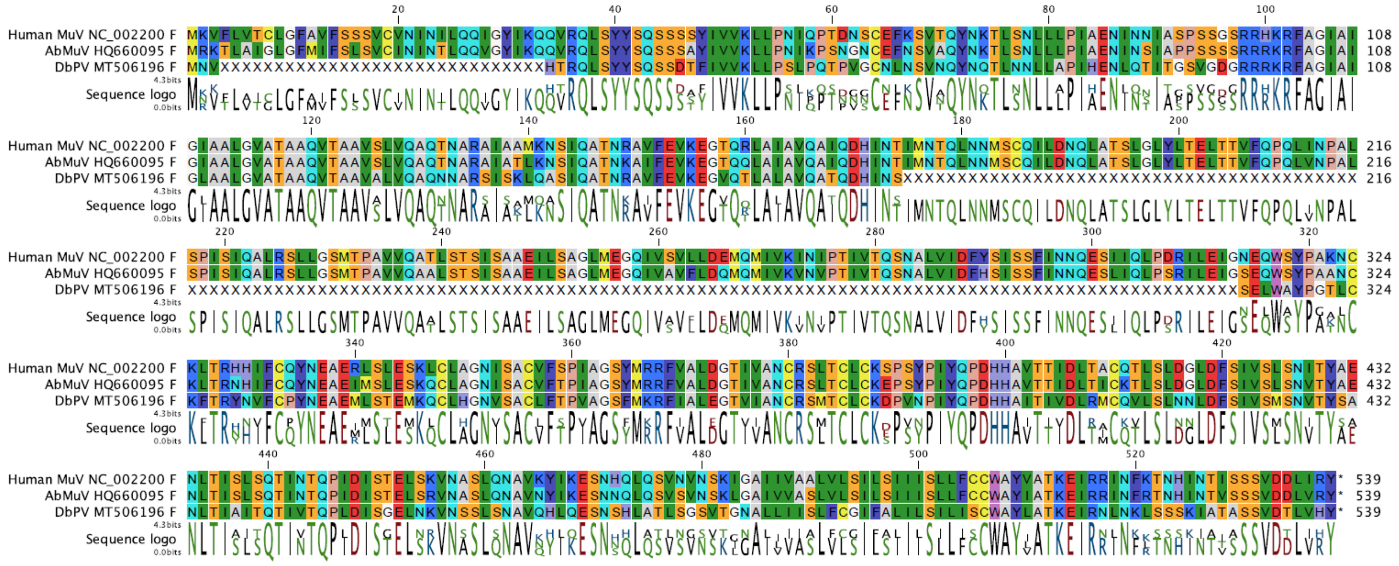

Supplemental Figure S3. Alignment of predicted F amino acid sequences for Human MuV, AbMuV, and DbPV. There were 30 and 139 aa long gaps in the predicted 538 aa sequence for F of DbPV. We identified the conservation of an important cleavage motif in F: AbMuV and DbPV encode a putative multibasic furin cleavage motif (RRRKR|F) that is conserved, see positions 98-103 (also highlighted in Figure 4) **[23,24]**. We also observed a lack of conservation among the amino acid positions 323 and 373 (EU370207) in the F aa sequence. These positions are significant because they are part of at least one neutralizing conformational epitope **[21]**.

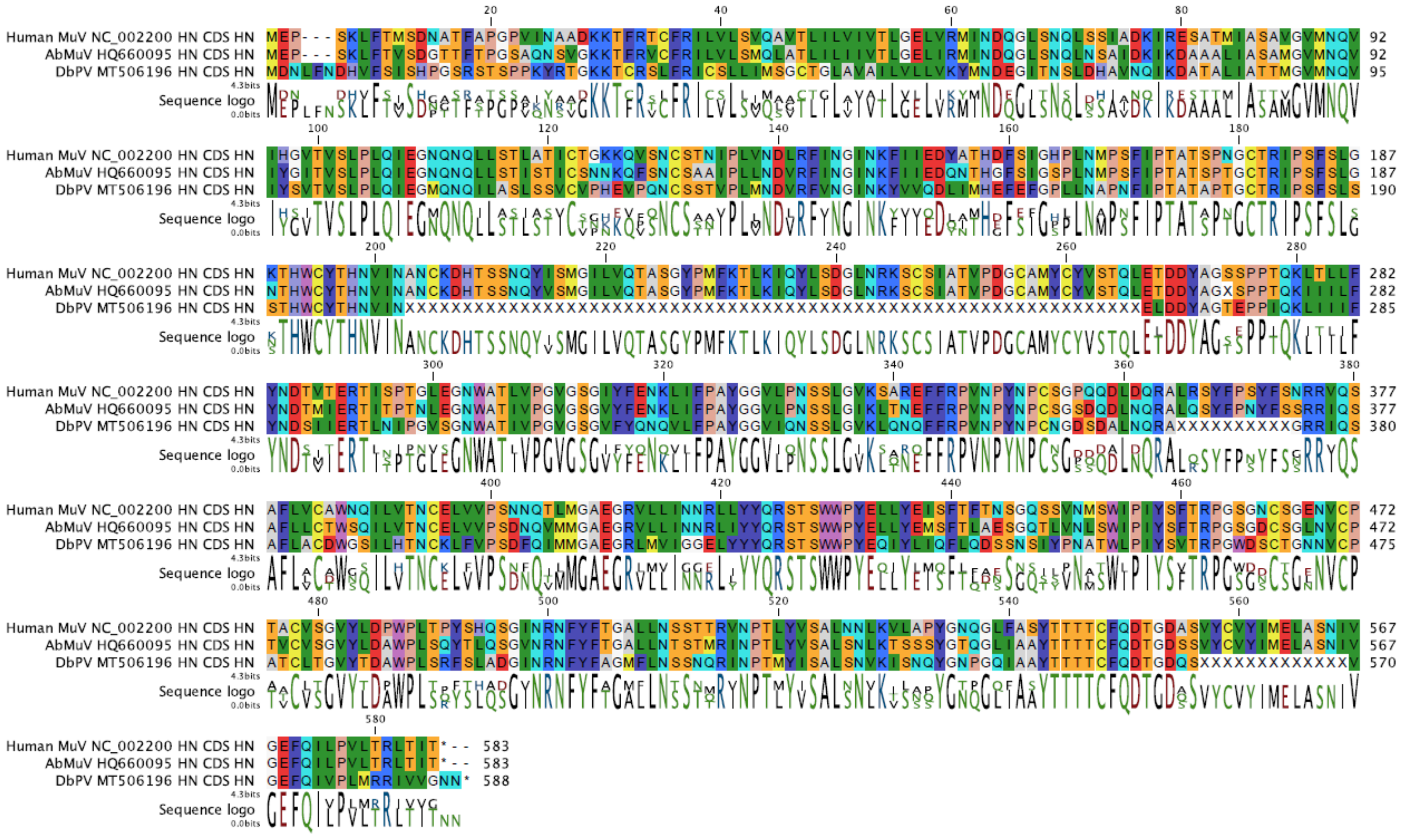

Supplemental Figure S4. Alignment of predicted HN amino acid sequences for Human MuV, AbMuV, and DbPV. The predicted 587 aa DbPV sequence for HN has 3 gaps that are 64, 10, and 13 aa long.
